# An In Vitro Study on the Shear Bond Strength of Feldspathic Porcelain to Nickel Chromium Alloy and Cobalt Chromium Alloy after Various Surface Treatments

**DOI:** 10.1155/2022/2557127

**Published:** 2022-05-30

**Authors:** Vignesh Kamath, Mayuri Kamath, Abhinav Bhargava, Thilak Shetty, Shobha J. Rodrigues, Umesh Y. Pai, Sharon Saldanha, M. Mahesh, Puneeth Hegde, Prashant Bajantri, Sandipan Mukherjee, Ann Sales

**Affiliations:** ^1^Department of Prosthodontics and Crown and Bridge, Manipal College of Dental Sciences, Mangalore, Manipal Academy of Higher Education, Manipal, Karnataka, India; ^2^Department of Prosthodontics Crown and Bridge, A. J Institute of Dental Sciences, Mangalore, Karnataka 575004, India; ^3^Department of Public Health Dentistry, SGT Dental College, Hospital & Research Institute, SGT University, Gurugram, India

## Abstract

**Background:**

To evaluate and compare the shear bond strength of feldspathic porcelain to four distinctively surface-treated Ni-Cr and Co-Cr alloys and to assess the impact of oxidation-heat treatment on porcelain to base metal alloy bond strength.

**Methods:**

40 specimens each of nickel-chromium alloy and cobalt-chromium alloy were cast. A total of four groups of specimens were created. Group I was surface-treated by sandblasting with 50 *μ*m alumina particles, Group II was surface-treated by sandblasting with 110 *μ*m alumina particles, Group III and Group IV were surface-treated with 250 *μ*m alumina particles. In Group IV, after sandblasting initially with 250 *μ*m alumina particles, the alloys were subjected to oxidation and resandblasting with 250 *μ*m alumina particles. Each of the specimen was coated with opaque and body porcelain and fired to a total thickness of 2 mm porcelain. A universal measuring machine was used to assess shear bond strength at a crosshead speed of 0.5 mm/min.

**Results:**

Two-way ANOVA followed by Tukey's post hoc test was used to assess the significant difference within the groups. Unpaired *t*-test was used for the intergroup comparison of the obtained data. The study showed that the size of the air abrasion particles used for sandblasting significantly influenced the porcelain to metal surface bond strength, with *p* value <0.001. The bond strength values of the two alloys tested showed no major variations. Result also showed that oxidation influences the metal-ceramic bond strength.

**Conclusions:**

The bond strength of the metal-ceramic interface is influenced by the alloy's surface treatment. The oxidation process impacts the bond strength of the metal-ceramic system.

## 1. Introduction

Porcelain-fused-to-metal restorations have been used in dentistry for ages because of their good clinical results, aesthetics, and longevity [1]. As the price of gold alloys upturned over time, the use of alternative alloys became more popular. Despite some drawbacks such as possible biologic risks, difficult handling, and the formation of chromic oxide, base metal alloys were favored over noble metal alloys due to superior mechanical properties, better rigidity, and lower cost [2].

The optimum relationship between the metal and the ceramic substructure is critical to the success of porcelain-fused-to-metal restoration [3]. Van der Waals forces, chemical bonding, and mechanical interlocking are believed to be involved in the bonding of porcelain to the alloy. The biochemical compatibility of alloy and porcelain is of utmost importance to withstand the mechanical stresses and forces directed towards its interface [4–6].

Currently, the most commonly used base metal alloy in clinical practice is Ni-Cr alloy with or without beryllium. However, long-term exposure to Ni and Be is concerned with possible damage to the health of the patient as well as professional [7, 8]. Hence, more biocompatible alloys must be substituted for Ni-Cr alloys. Co-Cr alloy is a good alternative to Ni-Cr alloys. Studies have shown that Co-Cr alloys are generally well tolerated and are more biocompatible to Ni-Cr alloys [9, 10]. However, metal-ceramic systems do not have all of their efficiency and properties fully defined.

The development of a chromic oxide layer is one drawback of base metal alloys, as a consequence of which the bond strength between the porcelain and metal is reduced [2, 11]. During the oxidation-heat treatment of metal, trapped gasses are eliminated, surface impurities are removed, and a metal oxide layer is formed. This originally formed oxide layer is then dissolved by the porcelain during the firing process, leading to the formation of the metal interface, essential for the development of a metal-ceramic bond [12].

Several methods and techniques have been tried over the years to improve the wettability of porcelain to alloy. Surface treatment of alloys is one of the techniques, and Al_2_O_3_ particles are most widely used for this purpose. It has been shown that sandblasting the alloy surface increases the surface energy and decreases the surface tension of the alloy, resulting in increased wetting of the alloy by the porcelain [10, 12, 13].

Constant evaluation of metal-ceramic bond strength has become important due to continuous technological advancement and increased day-to-day availability of all-ceramic systems in the industry. Hence, the present study was carried out to determine the shear bond strength of Co-Cr and Ni-Cr base metal alloys at the metal-ceramic interface after subjecting them to surface treatment with Al_2_O_3_ particles of various sizes. The study also aimed to analyze how oxidation-heat treatment affected porcelain to a base metal alloy bond strength.

## 2. Materials and Methods

### 2.1. Preparation of the Test Specimen

A silicone mold (3M ESPE Vinyl Polysiloxane Putty Impression Material, Neuss, Germany) was prepared from a machined stainless steel die which was fabricated to be 10 mm in width, 10 mm in breadth, and 1 mm in height. A custom silicone mold was used to create a total of 80 blocks of inlay casting wax (Kronenwachs, BEGO, Germany). Set wax patterns were removed from the mold and cast. The induction casting system (FORNAX T, BEGO, Germany) was used to cast base metal alloy pellets, 40 samples each of Ni-Cr (Wiron 99, BEGO, Germany) and Co-Cr (Wironit, BEGO, Germany) alloy. After divesting, sprues were removed and test specimens were cleaned and were subjected to finishing procedures.

### 2.2. Grouping of Samples

Collected specimens were categorized into 4 groups, each with 10 specimens. Sample size for this study was determined based on an earlier study [12] using the following equation:

(1) $$\begin{array}{c} n=z^{2}\times \frac{\sigma^{2}}{e^{2}}, \end{array}$$

where *n* = number of specimens in each group, *z* = critical value (3.030), *e* = error due to measurement (3.92), and *σ* = standard deviation (4.09).

Groups I (Control), II, III, and IV of each base metal alloy were subjected to air abrasion with Al_2_O_3_ particles (Hinrivest, Confident Sales, Karnataka, India) of various sizes. Two thin coats of opaque feldspathic porcelain were brush-coated onto each specimen and fired to a temperature of 950°C in a calibrated porcelain vacuum furnace (Programat P300 G2, Ivoclar Vivadent, Nagasaki, Japan). This was followed by the application of body porcelain (VITA VMK Master, Bad Sackingen, Germany) over the set opaque layer to achieve a thickness of 2 mm [12, 14]. The specimens were fired at 930°C under vacuum. A Vernier Caliper was used to measure the thickness of the ceramic layer. Glazing was completed at 910°C in the porcelain vacuum furnace [12, 14].

### 2.3. Testing of Shear Bond Strength of Specimen

**Figure 1:**
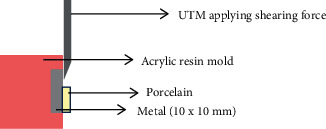
Shear bond strength tested using universal testing machine after fabrication of acrylic resin mold.

Each specimen was encased in a mold made of acrylic resin before being attached to the universal testing machine's shear test jig (Tinius Olsen, Philadelphia, USA). Compressive force was applied at metal-to-porcelain interface. The load was applied at a speed of 0.5 mm/min to that particular specimen until the adhesive fracture occurred [2, 7, 11, 12, 15], and the load readings were recorded in megapascal as shown in Figure 1. The same procedure was followed for each of the 80 specimens.

### 2.4. Statistical Analysis

Readings were subjected to appropriate statistical analysis. The data were checked for normality using the Kolmogorov–Smirnov test and the Shapiro–Wilk test. The data showed a normal distribution. ANOVA (analysis of variance) test followed by Tukey's post hoc test was used to determine if there was a substantial difference within the groups. The unpaired *t*-test was employed for intergroup comparison.

## 3. Result

Shear bond strength mean and standard deviation values of Ni-Cr alloy samples obtained using ANOVA are shown in Table 1. Post hoc comparisons between groups based on shear bond strength of Ni-Cr alloy samples were obtained using post hoc Tukey's tests as shown in Table 2. Results obtained for alloy samples were very highly significant (*p* < 0.001). Group III had the highest shear bond strength, while Group I had the lowest shear bond strength.

Shear bond strength mean and standard deviation values of Co-Cr alloy samples obtained using ANOVA are shown in Table 3. Post hoc comparison between groups based on shear bond strength of Co-Cr alloy samples was obtained using post hoc Tukey's tests as shown in Table 4. Results obtained for alloy samples were very highly significant (*p* < 0.001). Group III had the highest shear bond strength, while Group I had the lowest shear bond strength. Results confirmed that the shear bond strength of the alloy-porcelain interface is affected by alumina grit size.

Results also showed that the shear bond strength of both the alloys was significantly reduced after sandblasting with alumina particles, followed by oxidation and sandblasting again.

**Figure 2:**
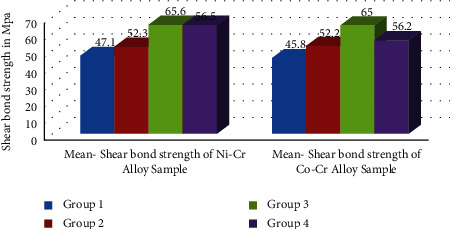
Intergroup comparison of shear bond strength of Ni-Cr and Co-Cr alloy samples.

**Figure 3:**
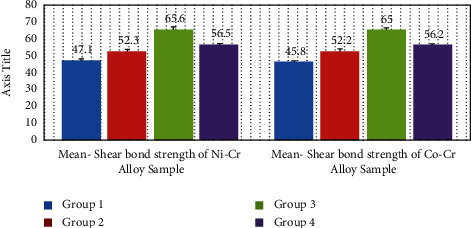
Standard deviation error bars.

An unpaired *t*-test was used for the intergroup comparison of the shear bond strength of the alloys as shown in Figure 2 and Table 5.  Figure 3 shows standard deviation error bars between the two alloy groups. Except for group I, there was no substantial difference in the shear bond strength of the alloys tested.

## 4. Discussion

The metal-ceramic bond interface is crucial to a restoration's functional and aesthetic performance. Many methods are proposed over the years to quantify such adhesion [3]. Surface treatment of alloys before porcelain firing is becoming the bottom of interest in various systems [16]. Thus this research was carried out to determine the shear strength of the porcelain to Ni-Cr and Co-Cr alloys when subjected to a variety of surface modifications, which are essential for the durability of metal-ceramic restorations.

The Ni-Cr alloy is the most extensively used alloy in metal-ceramic prosthesis. There have been concerns raised about toxic and allergenic elements, as well as the alloys' carcinogenic potential [17, 18]. Ni is one of the most likely reasons of allergic dermatitis, and it has been identified in studies as a component with greater allergenic and toxic effects when combined with Be. As a result, the Co-Cr alloys were regarded as safe clinical replacements with acceptable physical and mechanical properties [18–20].

A literature review has revealed that the airborne-particle abrasion of bonding surfaces increased metal surface energy by enhancing the wettability of the opaque ceramic and, as a result, the bond strength [11]. The particle size of the material used for surface treatment also influences the bond strength of the restoration [10, 12, 16]. Hence, different grit sizes Al_2_O_3_ were used for the surface treatment of the metal to see whether the particle size of Al_2_O_3_ impacts the metal-ceramic bond strength.

The oxide layer formed during the oxidation process influences the bond strength of the metal-ceramic system [21, 22]. The formation of metal oxides during the oxidation process varies depending on the alloy, the surface finishing technique, and the length of the oxidation process [23, 24]. Hence, in the present study, oxidation was done after sandblasting in group IV to judge whether the oxide layer initially produced before sandblasting was not the same as the oxide layer that was produced by sandblasting afterward and also to judge how it affected the shear bond strength.

The shear bond strength of the material significantly reduced after sandblasting with Al_2_O_3_ particles, preceded by oxidation and sandblasting again. The results also revealed no substantial difference in shear bond strength between Co-Cr and Ni-Cr alloys, indicating that Co-Cr alloy could be used clinically as an alternative to Ni-Cr alloy.

Many variables can have a significant influence on the shear bond strength of metal ceramics such as choice of alloys, surface roughness of alloy, contamination of alloy before porcelain firing, interatomic bonding between porcelain and the metal oxide, interatomic bonding across the oxide porcelain interface, type, and magnitude of residual stress in the veneering porcelain [25–27]. These concerns should be evaluated in future studies.

Other treatment alternatives that can be used to improve the bonding between the metal alloy and the porcelain are irradiation of alloy by the Nd : YAG laser, using milled sintered base metal alloy, oxidation of the alloy surface, using alloy primer, surface grinding of alloy, degassing, ultrasonic cleaning, and by means of the thermocycling procedure [13, 28–30]. Further research involving these options would provide a more complete understanding of the effect of alloy surface modifications on metal-ceramic bond strength.

Though metal-ceramic systems are selected due to their strength and versatility, newer metal-free crowns are increasingly being used in dental practice over the last four decades as an alternative for PFM crowns to overcome their aesthetic limitations; these crowns are usually made from different ceramic materials such as lithium disilicate, zirconia, leucite-reinforced glass, and glass-infiltrated alumina [14, 25, 31].

In vitro experiments also depend on a variety of factors that can influence the study's outcome. Thus, controlling all the external factors that may play a role in the result is difficult. Cohesive or adhesive failures occur in metal ceramics. Cohesive failures occur between the ceramic layers. Only adhesive failures were considered for the bond strength to be measured in this analysis. Though the laboratory studies guide in thorough selection of materials, further clinical trials should be encouraged to support the existing data and improve the clinical standards.

## 5. Conclusion

The bond strength of the metal-ceramic contact is influenced by surface treatment. The particle size of the material used for the surface treatment of the alloy greatly influenced the bond strength of the alloy to porcelain. In comparison to previous techniques, sandblasting casting alloys, followed by oxidation and sandblasting, decreased bond strength. This comparison is limited between specimens that have been sandblasted with 250 alumina particles. No significant difference was seen in shear bond strength between Ni-Cr and Co-Cr alloys aside from Group I.

## Figures and Tables

**Table 1:** Overall comparison of shear bond strength of Ni-Cr alloy samples using ANOVA.

	Mean	Standard deviation	Minimum	Maximum	*N*
Group 1	47.1	1.08	45.23	48.78	10
Group 2	52.3	1.43	50.08	54.6	10
Group 3	65.6	1.49	63.23	67.72	10
Group 4	56.5	0.71	55.3	57.6	10
*f* value	405.906				Total = 40
*p* value	0.001^*∗∗*^				

*p* < 0.05, significant; *p* < 0.01, highly significant; *p* < 0.001, very highly significant; *p* > 0.05, not significant.

**Table 2:** Post hoc comparison between groups based on shear bond strength of Ni-Cr alloy samples using post hoc Tukey's tests.

Group		Mean difference	Standard error	95% confidence interval		*p* value
				Lower bound	Upper bound	
Group 1	Group 2	−5.18	0.54719	−6.65	−3.7	0.001
Group 1	Group 3	−18.42		−19.89	−16.95	0.001
Group 1	Group 4	−9.39		−10.86	−7.91	0.001
Group 2	Group 3	−13.24		−14.71	−11.77	0.001
Group 2	Group 4	−4.21		−5.68	−2.73	0.001
Group 3	Group 4	9.03		7.55	10.5	0.001

*p* < 0.05, significant; *p* < 0.01, highly significant; *p* < 0.001, very highly significant; *p* > 0.05, not significant.

**Table 3:** Overall comparison of shear bond strength of Co-Cr alloy samples using ANOVA.

	Mean	Standard deviation	Minimum	Maximum	*N*
Group 1	45.8	1.18	44.06	47.82	10
Group 2	52.2	1.80	50.14	55.68	10
Group 3	65.0	1.54	61.23	66.56	10
Group 4	56.2	0.83	54.82	57.11	10
*f* value	333.714				Total = 40
*p* value	0.001^*∗∗*^				

*p* < 0.05, significant; *p* < 0.01, highly significant; *p* < 0.001, very highly significant; *p* > 0.05, not significant.

**Table 4:** Post hoc comparison between groups based on shear bond strength of Co-Cr alloy samples using post hoc Tukey's tests.

Group		Mean difference	Standard error	95% confidence interval		*p* value
				Lower bound	Upper bound	
Group 1	Group 2	−6.39	0.62279	−8.07	−4.71	0.001
Group 1	Group 3	−19.21		−20.89	−17.54	0.001
Group 1	Group 4	−10.39		−12.06	−8.71	0.001
Group 2	Group 3	−12.82		−14.5	−11.14	0.001
Group 2	Group 4	−3.99		−5.67	−2.31	0.001
Group 3	Group 4	8.82		7.15	10.5	0.001

*p* < 0.05, significant; *p* < 0.01, highly significant; *p* < 0.001, very highly significant; *p* > 0.05, not significant.

**Table 5:** Intergroup comparison of shear bond strength of Ni-Cr and Co-Cr alloy samples using unpaired *t*-test.

	Group	*N*	Mean	Standard deviation	*T*	df	Sig. (2-tailed)
One	Ni-Cr	10	47.1	1.08	2.671	18	0.016^*∗*^
	Co-Cr	10	45.8	1.18			

Two	Ni-Cr	10	52.3	1.43	0.2	18	0.844
	Co-Cr	10	52.2	1.80			

Three	Ni-Cr	10	65.6	1.49	0.831	18	0.417
	Co-Cr	10	65.0	1.54			

Four	Ni-Cr	10	56.5	0.71	1.031	18	0.316
	Co-Cr	10	56.2	0.83			

*p* < 0.05, significant; *p* < 0.01, highly significant; *p* < 0.001, very highly significant; *p* > 0.05, not significant.

## Data Availability

Data are available from the corresponding author on reasonable request.
